# Zika virus infection suppresses CYP24A1 and CAMP expression in human monocytes

**DOI:** 10.1007/s00705-024-06050-2

**Published:** 2024-06-06

**Authors:** Lady Johana Hernández-Sarmiento, Juan Felipe Valdés-López, Silvio Urcuqui-Inchima

**Affiliations:** Grupo Inmunovirología, Facultad de Medicina, Universidad de Antioquia UdeA, Calle 70 No. 52-21, Medellín, Colombia

## Abstract

Monocytes are the primary targets of Zika virus (ZIKV) and are associated with ZIKV pathogenesis. Currently, there is no effective treatment for ZIKV infection. It is known that 1,25-dihydroxy vitamin D_3_ (VitD3) has strong antiviral activity in dengue virus-infected macrophages, but it is unknown whether VitD3 inhibits ZIKV infection in monocytes. We investigated the relationship between ZIKV infection and the expression of genes of the VitD3 pathway, as well as the inflammatory response of infected monocytes *in vitro*. ZIKV replication was evaluated using a plaque assay, and VitD3 pathway gene expression was analyzed by RT-qPCR. Pro-inflammatory cytokines/chemokines were quantified using ELISA. We found that VitD3 did not suppress ZIKV replication. The results showed a significant decrease in the expression of vitamin D3 receptor (VDR), cytochrome P450 family 24 subfamily A member 1 (CYP24A1), and cathelicidin antimicrobial peptide (CAMP) genes upon ZIKV infection. Treatment with VitD3 was unable to down-modulate production of pro-inflammatory cytokines, except TNF-α, and chemokines. This suggests that ZIKV infection inhibits the expression of VitD3 pathway genes, thereby preventing VitD3-dependent inhibition of viral replication and the inflammatory response. This is the first study to examine the effects of VitD3 in the context of ZIKV infection, and it has important implications for the role of VitD3 in the control of viral replication and inflammatory responses during monocyte infection.

**Supplementary Information:**

The online version contains supplementary material available at 10.1007/s00705-024-06050-2.

## Introduction

Zika virus (ZIKV) infection is a public health issue of worldwide concern [1]. ZIKV is an arbovirus belonging to family *Flaviviridae*, genus *Orthoflavivirus* [2]. It is a blood-borne pathogen that is transmitted through the bite of infected mosquitoes of the genus *Aedes*, but it can also be transmitted through human-to-human contact via sexual contact, blood transfusion, or vertical transmission from pregnant mothers to fetuses [3–5]. While the initial documentation of the virus traces back to the early 1950s, the emergence of the virus in the Americas, specifically in Brazil and Colombia [6, 7], was a turning point in ZIKV research because the virus was found to be associated with severe complications, including microcephaly and teratogenesis in newborns, as well as Guillain-Barré syndrome and Alice in Wonderland syndrome in adults [8–10].

Infection with ZIKV, the causal agent of Zika fever (ZIKF), leads to a temporary febrile illness that affects around 20% of individuals who contract the virus [11]. During the acute phase of ZIKV infection, patients have elevated serum levels of pro- and anti-inflammatory cytokines, including tumor necrosis factor alpha (TNFα), interleukin (IL) 1β (IL1β), IL2, IL4, IL6, IL9, IL10, IL13, and IL17. These cytokines have been linked to the severity of the disease [12–14].

Monocytes, which are phagocytic cells of the innate immune system, have a significant impact on the control and immunopathogenesis of viral infections. It has been suggested that monocytes can be a double-edged sword in viral infections, acting as initiators of the initial wave of inflammatory response but also as vessels for viral spread [15], acting as “Trojan horses” [16]. This phenomenon has been reported previously for ZIKV [17] and Visna virus [18]. Furthermore, circulating monocytes have been identified as the primary target cells of ZIKV infection in pediatric patients, as well as *in vitro* and *ex vivo* [17, 19].

Although ZIKV represents a significant global public health concern, there is currently a lack of effective treatments or vaccines to combat the spread of ZIKV [20, 21]. Thus, it is necessary to explore new alternative approaches to control the progression of ZIKV infection. One potential strategy is the use of antiviral molecules that also have immunomodulatory properties, such as 1,25-dihydroxyvitamin D3 (1,25(OH)_2_D3; VitD3), also known as calcitriol. It is the active form of vitamin D, which has been shown to possess immunomodulatory and antiviral properties against viral infections in humans (Reviewed in: [22]). For instance, the susceptibility of monocyte-derived dendritic cells (MDDCs) and monocyte-derived macrophages (MDMs) to DENV infection *in vitro* was reduced when healthy volunteers were given a supplement of 4000 international units of calcitriol per day [23–25]. Moreover, decreased production of pro-inflammatory cytokines and increased IL10 secretion were observed. Similar results have been reported in infections by influenza A virus [26], respiratory syncytial virus (RSV) [27], human immunodeficiency virus 1 (HIV-1) [28–30], and SARS-CoV-2 [31, 32]. Furthermore, vitamin D deficiency leads to altered immune function that can impact the response to viral infections. For example, a connection has been established between vitamin D deficiency and the severity of COVID-19 disease [33]. Mirza et al. [2022] reported that dengue fever patients coinfected with *Helicobacter pylori* who had insufficient vitamin D levels were much more susceptible to infection by all four dengue virus (DENV) serotypes [34], as well as infection by HIV-1 [35, 36].

The biologically active form of vitamin D can be synthesized from the skin as pre-vitamin D3 in response to UV radiation by converting 7-dehydrocholesterol or can be obtained through the absorption of dietary components such as ergocalciferol (vitamin D_2_). Both forms require two hydroxylation steps for activation. The initial hydroxylation occurs in the liver, catalyzed by mitochondrial and microsomal vitamin D 25-hydroxylase or the enzymes CYP2R1 and CYP27A1, resulting in the formation of calcidiol or 25(OH)D [37]. Calcidiol, which remains in the bloodstream for an extended period, is used as a marker to assess serum vitamin D levels (reviewed in [38]). Subsequently, calcidiol undergoes hydroxylation in the kidney through the enzymatic activity of 1-alpha-hydroxylase (CYP27B1), resulting in the production of vitamin D3 [39], which binds the vitamin D receptor (VDR) in the cell membrane, which is responsible for the biological activity of vitamin D. VDR is expressed in different types of cells, including T cells, monocytes, and macrophages. The vitamin D-VDR complex is translocated to the nucleus, where it interacts with the nuclear retinoic acid X receptor (RXR) to form a heterodimer that functions as a transcription factor for vitamin D response elements (VDREs) on target genes. Finally, the CYP24A1 enzyme inactivates calcitriol and calcidiol through successive hydroxylation reactions. VDR, in turn, regulates the expression of primary VitD3 target genes such as cathelicidin antimicrobial peptide (CAMP) and CYP24A, which are involved in VitD3 catabolism [40].

Human monocytes were among the first immune cells shown to express VDR [41], making them targets of VitD3 and enhancing their antimicrobial properties [42, 43]. Other studies showed an increase in CAMP gene transcription in monocytes stimulated with VitD3, resulting in higher production of the active form of the antimicrobial peptide LL-37 [44]. The increase in LL-37 has been shown to enhance monocyte function and suggests that LL-37 improves its activity against viral infections. Previously, we reported the induction of a pro-inflammatory and antiviral response in ZIKV-infected monocytes [45], although the immunomodulatory and antiviral role of VitD3 treatment in ZIKV-infected monocytes has not been reported. Here, we assess the impact of VitD3 treatment on ZIKV replication, the expression of genes encoding TLRs, pro-inflammatory and antiviral factors, and the VitD3 pathway in ZIKV-infected monocytes.

## Materials and methods

### Ethics statement

As reported previously [45, 46], the individual enrollment and sample collection protocols were authorized by the Committee of Bioethics Research at Sede de Investigación Universitaria, Universidad de Antioquia (Medellín, Colombia). Prior to participation, all individuals provided informed consent by signing a form, and the study was conducted in accordance with the principles outlined in the Declaration of Helsinki. This study involved the participation of three to four healthy donors.

## Cells lines, ZIKV stock production, and virus titration

*Ae. albopictus*-derived C6/36-HT cells (ATCC) were grown in Leibovitz’s L-15 medium (L-15; Sigma-Aldrich) supplemented with 5% heat-inactivated fetal bovine serum (FBS; Gibco, Thermo Fisher Scientific, Massachusetts, USA) and 1% antibiotic-antimycotic solution (Corning, New York, USA) and incubated at 34°C in cell culture flasks at a density of 1 × 10^5^-1 × 10^6^ cells/mL. ZIKV Colombia strain (GenBank no. MH179341.1) isolated from mosquitoes (kindly provided by Professor Blanco P. Universidad de Sucre, Colombia) was obtained by growth in C6/36-HT cells as reported previously [45]. Virus culture supernatants were stored at -80°C and titrated by plaque assay on BHK-21 cells (clone 15, ATCC) as described previously [45]. Briefly, 5 × 10^4^ BHK-21 cells per well were seeded in 48-well dishes in Dulbecco’s modified Eagle medium (DMEM; Sigma-Aldrich, St. Louis, USA) supplemented with 2% FBS, 0.3% (v/v) NaHCO_3_, and 1% (v/v) antibiotic-antimycotic solution, and incubated at 37°C and 5% CO_2_ for 24 hours. The cells were then infected with serial dilutions of culture supernatants for 90 minutes, after which plaque assay medium (2% FBS, 1% HEPES [Sigma-Aldrich], 3% (v/v) sodium carboxymethyl cellulose [Sigma-Aldrich], and 2X DMEM medium [DMEM powder, Sigma-Aldrich]) was added. BHK-21 cells were incubated at 37°C and 5% CO_2_ for 4 days. Then, lysis plaques were stained using a crystal violet solution (2% crystal violet, 1.5% formaldehyde). The virus titer was determined to be 1.1 × 10^7^ PFU/mL.

## Culture of primary human monocytes and treatment with vitamin D3

Human peripheral blood mononuclear cells (PBMCs) were obtained from blood samples of healthy donors. The PBMCs were mixed with 2% (v/v) EDTA and isolated using a density gradient with Lymphoprep (STEMCELL Technologies Inc., Vancouver, Canada) through centrifugation at 850 × *g* for 21 min as described previously [47]. Platelets were depleted by washing three times with phosphate-buffered saline (PBS; Sigma-Aldrich) at 250 × *g* for 10 min, and the percentage of CD14-positive cells was determined by flow cytometry. To obtain monocytes, 24-well plastic plates were scratched with a 1000-μL pipette tip, seeded with 5 × 10^5^ CD14-positive cells per well, allowed to adhere for 2 h in RPMI-1640 medium (Sigma-Aldrich) supplemented with 0.5% autologous serum, 0.3% NaCO_3_, and 4 mM L-glutamine, and cultured at 37°C and 5% CO_2_. Non-adherent cells were removed by washing twice with PBS, and monocytes were cultured in RPMI-1640 medium supplemented with 10% FBS, 0.3% NaHCO_3_, 4 mM L-glutamine, and 1% antibiotic-antimycotic solution (complete medium), as described previously [45]. Human monocytes were cultured in the absence (Mon) or presence (VitD3-Mon) of 1 nM VitD3 (Sigma Aldrich, USA) and incubated at 37°C/5% CO_2_ overnight.

## *In vitro* infection of monocytes

Monocytes were divided into two groups, one of which was cultured in presence of VitD3 (VitD3-Mon), and the other without VitD3 (Mon). After 12 hours of culture, both groups were infected with ZIKV at a multiplicity of infection (MOI) of 5 (ZIKV-Mon and ZIKV-VitD3-Mon, respectively) in serum-free RPMI-1640. Samples were incubated at 37°C for 1.5 h. Then, the cells were washed with PBS to remove the unbound virus, and fresh complete medium with or without VitD3 was added. Both Mon and VitD3-Mon were included as uninfected controls. Cells were incubated at 37°C/5% CO_2_, and culture supernatants and cell lysates were collected at 6, 12, 24, 48, and 72 hours postinfection (hpi) and stored at -80°C.

## Plaque assay

Culture supernatants of ZIKV-infected monocytes with or without VitD3 treatment were titrated by plaque assay on BHK-21 cells (clone 15, ATCC) as described above.

## Real-time PCR for TLRs, VDR, CYP24A1, and CAMP

Total RNA was extracted using a tQuick-RNA Miniprep Kit (Zymo Research, USA), following the manufacturer´s instructions. The RNA concentration was determined using a NanoDrop 1000 spectrophotometer (Thermo Scientific, Wilmington, DE). cDNA synthesis was performed using a RevertAid Minus First Strand cDNA Synthesis Kit (Thermo Scientific, NH, USA), following the manufacturer´s instructions. The levels of TLR7, TLR8, VDR, CYP24A1, CAMP, and glyceraldehyde-3-phosphate dehydrogenase (GAPDH) mRNA in monocytes were determined by RT-qPCR, using previously reported gene-specific primers [48, 49]. Bio-Rad CFX Manager was used to determine the cycle threshold (Ct) for each sample, using a regression fit in the linear phase of the PCR amplification curve. RT-qPCR was carried out using the SYBR-Green system (Invitrogen, Oregon, USA), and the ΔΔCt method was used to determine the fold change (FC) [45, 49]. The relative quantification (FC) of each mRNA was normalized to the internal control GAPDH, and the uninfected Mon (control). |Log2 FC| > 0.6 was used as the threshold for a significant difference in gene expression.

## Quantification of cytokines and chemokines

The ELISA MAX Deluxe Set Human (BD Biosciences, San Jose, CA, USA) was used for quantification of TNFα, IL1β, IL6, IL10, CCL2, CCL5, and CXCL8/IL8 in cell culture supernatants, following the manufacturer´s instructions. The detection limit was 0.5-10 pg/mL.

## Statistical analysis

GraphPad Prism 8.0.1 (GraphPad Software Inc., San Diego, CA, USA) was used for statistical analysis. The Shapiro-Wilks test was performed to assess the normality of the data. The specific statistical tests employed in the analysis are indicated in the figure legends. Data are represented as the mean ± SEM. Significant results were defined as follows: *, *p* < 0.05; **, *p* < 0.01; ***, *p* < 0.001.

## Results

### Lack of effect of VitD3 on ZIKV replication in human monocytes

**Fig. 1:**
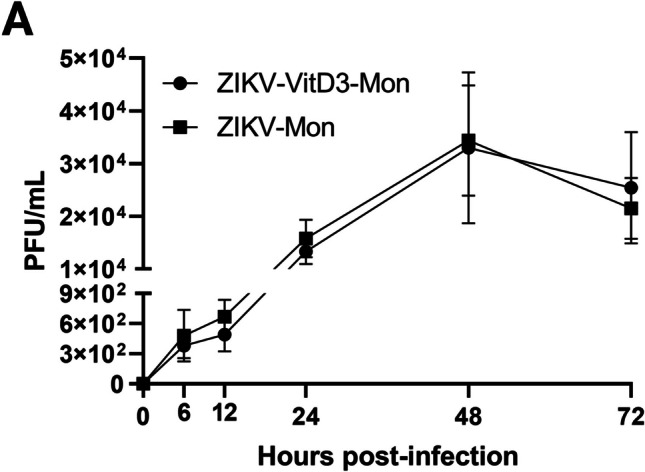
Effect of VitD3 treatment on ZIKV growth kinetic in monocytes. Primary human monocytes treated (VitD3-Mon) or not treated (Mon) with VitD3 were infected with ZIKV. Cell culture supernatants were harvested at the indicated time points, and infectious virus particles were quantitated as plaque-forming units/mL (PFU/mL). Data are represented as the mean ± SEM (*n* = 4). A repeated measures ANOVA test was performed. Significant results were defined as *p* < 0.05 (*)

We reported recently that ZIKV replicates in human monocytes [45], and in this study, we examined the potential of VitD3 to control ZIKV replication. As shown in Figure 1, we found that VitD3 treatment with 1.0 nM does not have a significant influence on ZIKV replication, as assessed by estimating the production of infectious virus particles by plaque assay at different time points (6 to 72 hpi). Similar results were obtained in a separate experiment using 0.1, 1.0, and 10 nM VitD3 (Supplementary Fig. S1A). We therefore used 1.0 nM VitD3 in subsequent experiments.

## Downregulation of VitD3 signaling pathway genes in ZIKV-infected monocytes

**Fig. 2:**
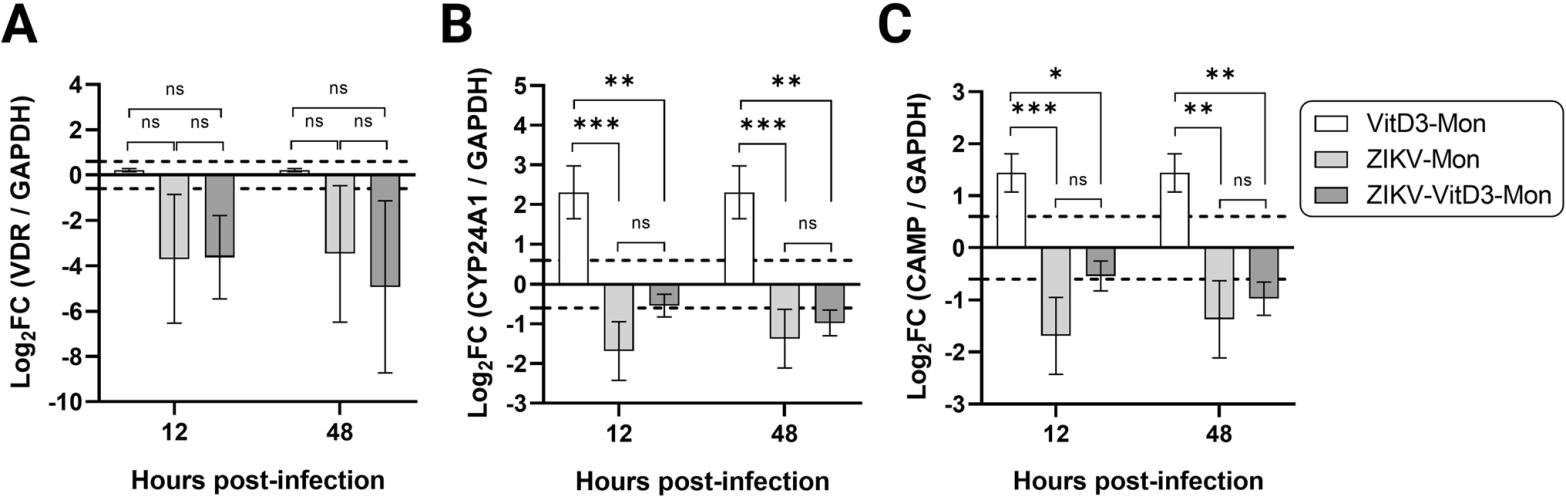
mRNA expression of *VDR*, *CYP24A1*, and *CAMP* during ZIKV replication in VitD3-treated (VitD3-Mon) and untreated monocytes*.* VitD3-Mon and Mon were infected with ZIKV (ZIKV-VitD3-Mon and ZIKV-Mon, respectively) and harvested at the indicated time points. mRNA expression of *VDR* (**A**), *CYP24A1* (**B**), and *CAMP* (**C**) in ZIKV-Mon and ZIKV-VitD3-Mon was analyzed by RT-qPCR. The ΔΔCt (threshold) method was used to determine the fold change (FC). The relative quantification (FC) of each mRNA was normalized to the housekeeping gene GAPDH, and uninfected monocytes (control). A log_2_ FC of 0.6 and −0.6 was used as the threshold for up- and downregulation of gene expression, respectively (dotted lines). A two-way repeated measures ANOVA test was performed (*n* = 3). ***, *p* = 0.001; ** *p* = 0.01; * *p* = 0.05; ns, not significant

The effect of ZIKV infection on the VitD3 system of monocytes was investigated by determining the relative levels of expression of VDR, CYP24A1, and CAMP mRNA by RT-qPCR. As illustrated in Figure 2A, VDR expression was not affected by VitD3 treatment, but a decreased level of transcription was observed at 12 and 48 hpi in ZIKV-infected cells, both with and without VitD3 treatment. At a concentration of 1 nM, VitD3 significantly upregulated the expression of CYP24A1 and CAMP at both 12 and 48 hours after treatment. However, in infected cells, significantly lower levels of CYP24A1 and CAMP expression were observed at 12 and 48 hpi with or without VitD3 treatment when compared to uninfected VitD3-treated cells (Fig. 2B and C). However, the inhibitory effect of ZIKV infection on CYP24A1 expression was less pronounced in the presence of VitD3 at both 12 hpi (*p* = 0.0005 and 0.0058, respectively) and 48 hpi (*p* = 0.001 and 0.0023, respectively) (Fig. 2B). Likewise, the inhibitory effect of ZIKV infection on CAMP expression was less pronounced in the presence of VitD3 at 12 hpi (*p* = 0.001 and 0.0178 respectively) (Fig. 2C), but the levels of CAMP were similar at 48 hpi (*p* = 0.0021 and 0.0057, respectively). The results show that ZIKV infection suppresses the expression of VDR, CYP24A1, and CAMP in monocytes, suggesting an alteration of the VitD3 signaling pathway.

## Effect of VitD3 treatment on TLR7 and TLR8 in ZIKV-infected monocytes

**Fig. 3:**
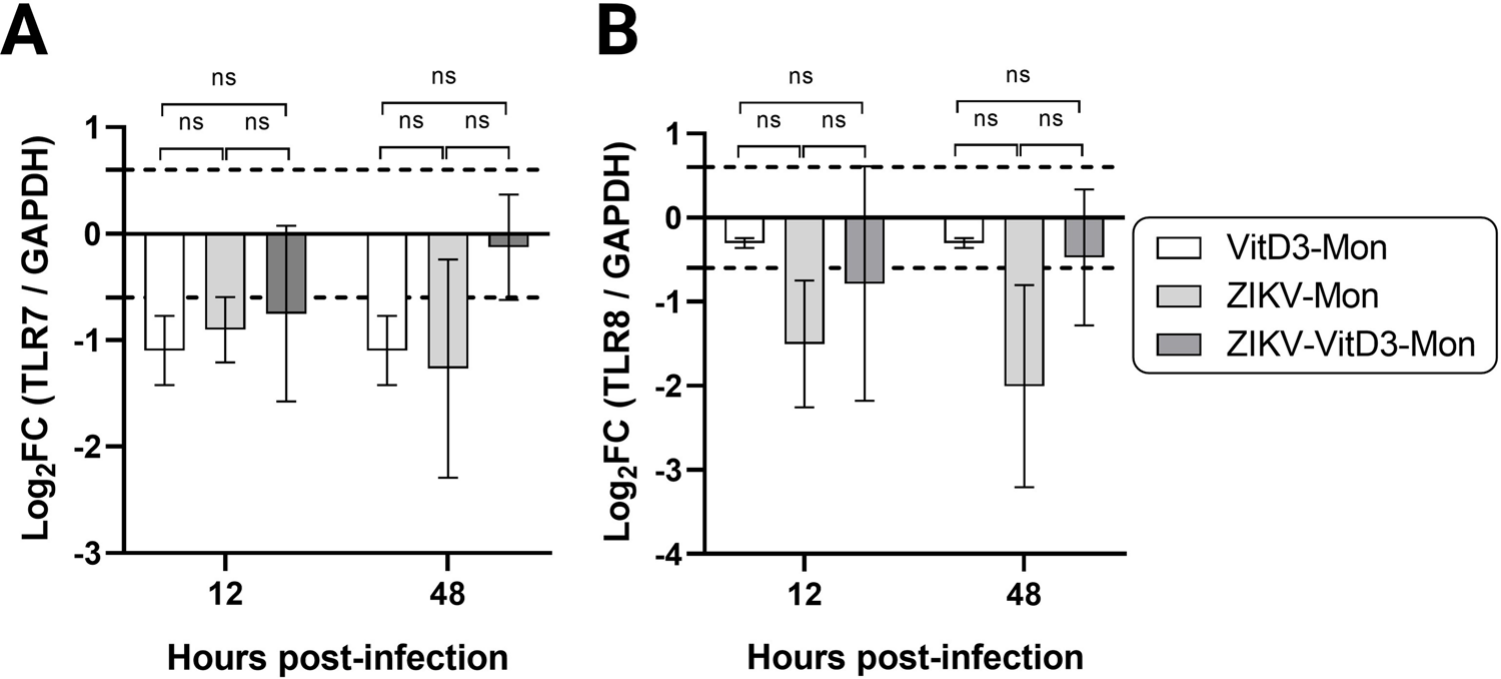
mRNA expression of *TLR7* and *TLR8* during ZIKV replication in VitD3-treated (VitD3-Mon) and untreated monocytes. VitD3-Mon and Mon were infected with ZIKV (ZIKV-VitD3-Mon and ZIKV-Mon, respectively) and harvested at the indicated time points. mRNA expression of *TLR7* (**A**) and *TLR8* (**B**) in ZIKV-Mon and ZIKV-VitD3-Mon was analyzed by RT-qPCR. The ΔΔCt (threshold) method was used to determine the fold change (FC). The relative quantification (FC) of each mRNA was normalized to the housekeeping gene GAPDH and uninfected monocytes (control). A log_2_FC of 0.6 and −0.6 was used as the threshold for up- and downregulation of gene expression, respectively (dotted lines). A two-way repeated measures ANOVA test was performed (*n* = 3). ***, *p* = 0.001; **, *p* = 0.01; *, *p* = 0.05; ns, not significant

Da Silva et al. [12] reported that patients with ZIKV infection show reduced expression of TLR8. It has also been shown that the TLR7/8 agonist R848 restricts the replication of ZIKV through induction of interferon-stimulated genes [50]. We therefore quantified the expression of TLR7 and TLR8 mRNA, which play a crucial role in detecting ZIKV RNA in the cell [50, 51], and found no significant effect of ZIKV infection in the presence or absence of VitD3 on the expression of either TLR7 and TLR8 when compared to uninfected VitD3-treated monocytes (Fig. 3A and B).

## Effect of VitD3 treatment on the inflammatory response in ZIKV-infected monocytes

**Fig. 4:**
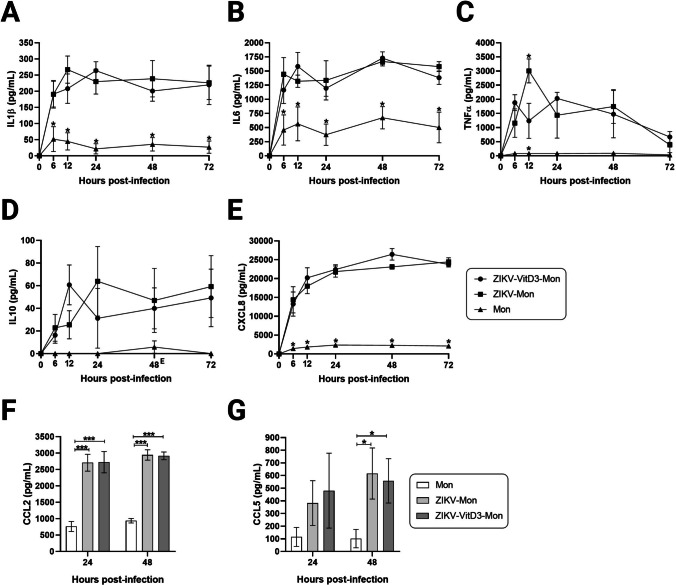
Production of cytokines and chemokines in VitD3-treated and untreated monocytes. VitD3-Mon and Mon were infected with ZIKV (ZIKV-VitD3-Mon and ZIKV-Mon, respectively) and harvested at the indicated time points. Uninfected monocytes were used as a control. The levels of IL1β (**A**), IL6 (**B**), TNFα (**C**), IL10 (**D**), CXCL8/IL8 (**E**), CCL2 (**F**), and CCL5 (**G**) were determined by ELISA. Data are represented as the mean ± SEM. A repeated measures ANOVA test was performed (*n* = 3). ***, *p* = 0.001; **, *p* = 0.01; *, *p* = 0.05

In ZIKV-infected monocytes (ZIKV-Mon), the production of IL1β, IL6, and TNFα, reached its peak at 12, 48, and 12 hpi, respectively, compared to the control (Fig. 4A, B, and C). This production was sustained over time. In contrast, in ZIKV-infected monocytes treated with VitD3 (ZIKV-VitD3-Mon), the production of IL1β, IL6, and TNFα reached its peak at 24, 48, and 24 hpi, respectively, compared to the control (Fig. 4A, B, and C). Considering that 1.0 nM VitD3 did not significantly decrease the production of TNFα and IL6, we evaluated the effect of 0.1, 1.0, and 10 nM of VitD3 to determine whether modulation of the inflammatory response is dose-dependent. No significant effect on the production of the two cytokines was observed, regardless of the VitD3 concentration used (Supplementary Fig. 1B and C). This suggests that VitD3 may have a limited effect on the secretion of pro-inflammatory cytokines during ZIKV infection in monocytes. Further research is needed to fully understand the effects of VitD3 on the immune response to ZIKV infection.

Similarly, ZIKV infection induced the production of IL10, with a peak at 24 hpi, in ZIKV-Mon, while in ZIKV-VD3-Mon, a peak was observed at 12 hpi (Fig. 4D). Although the addition of VitD3 resulted in a slight decrease in the IL10 level at 24 hpi, no significant difference was observed when compared to ZIKV-Mon (Fig. 4D). Furthermore, treatment with VitD3 had no effect on the production of the chemokines CXCL8/IL8, CCL2, and CCL5, in ZIKV-VitD3-Mon when compared to ZIKV-Mon (Fig. 4E, F, and G). However, both ZIKV-Mon and ZIKV-VitD3-Mon exhibited significantly increased IL1β, IL6, CXCL8/IL8, CCL2, and CCL5 production when compared to the control (uninfected Mon; Fig. 4). Together, these findings indicated that treatment of monocytes with VitD3 did not influence the inflammatory response to ZIKV infection.

## Discussion

Monocytes represent 10% of circulating leukocytes in humans. These immune cells, originating from the bone marrow, are released into the bloodstream and migrate to various tissues during viral infections and inflammation. Once in the tissues, they undergo differentiation into either macrophages or dendritic cells. Monocytes are the primary cellular target of ZIKV infection in humans, and Michlmayr et al. [19] and Foo et al. [52] found that they constitute about 84% of the *in vitro*-infected PBMCs and are linked to ZIKV pathogenesis. In our previous report, we documented an increase in the production of infectious ZIKV particles in human monocytes between 24 and 72 hpi, reaching a peak of viral replication at 48 hpi [45]. ZIKV infection has been shown to activate intracellular TLRs such as TLR3 [5, 53, 54] and TLR7/8 [50, 51] and to promote a strong pro-inflammatory and antiviral response. The ZIKV replication cycle has been extensively studied using cell lines or IFNAR-/- mice [55]. The anti-inflammatory and antiviral effects of VitD3 are well documented for various virus infections [23, 56–58], but its ability to modulate inflammatory responses and antiviral activity during ZIKV infection in monocytes has not been investigated. In this study, we elucidated the role of VitD3 in ZIKV infection in primary human monocytes and found that treatment with VitD3 did not lead to a decrease in the production of infectious viral particles. We also investigated whether ZIKV infection of monocytes decreased the expression of VitD3 signaling genes during virus infection. We measured the mRNA expression levels of VDR, which is essential for the biological activity of VitD3 [59], CYP24A1, which plays a role in inactivating calcitriol and calcidiol through a series of successive hydroxylation reactions [60], and CAMP, which is involved in mounting an immune response against a wide range of pathogenic microorganisms [61]. We found that ZIKV infection leads to decreased VDR mRNA expression. This might explain the lack of an effect of VitD3 on ZIKV replication in monocytes. Our findings are consistent with previous reports indicating that HIV-1 can impair innate immune defenses by downregulating the VDR pathway [62, 63]. We suggest that downregulation of VDR expression could potentially decrease vitamin D3 signaling, as has been observed in monocytes and macrophages infected with chikungunya virus (CHIKV) and treated with calcitriol [49].

The levels of CYP24A1 and CAMP mRNA were significantly lower at 12 and 48 hpi, two critical time points of ZIKV infection, in ZIKV-Mon and ZIKV-VitD3-Mon when compared to VitD3-Mon. The observed downregulation of CYP24A1 during ZIKV infection may further indicate specific viral interference with VDR gene expression. Similar to what has been observed previously in cytomegalovirus infections [64], our findings might indicate not only downregulation of VDR gene expression during ZIKV replication but also a reduction in the responsiveness of monocytes to VD3 treatment as a result of inhibition of the negative feedback loop caused by the relative deficiency of VDR-associated VitD3 [49]. This could provide an explanation for the lack of a decrease in the production of infectious ZIKV particles in ZIKV-VD3-Mon. Furthermore, genetic variations in the CYP24A1 locus have been associated with an increased risk of VitD3 insufficiency [65]. CYP24A1, a VDR target gene, plays a role in regulating the breakdown of VitD3 by converting both 25-OH-VD3 and 1α,25-(OH)2VD3 to 24-hydroxylated products, which are then eliminated through established pathways [66].

Virus-infected cells that exhibit low expression of vitamin D3 signaling genes can cause a reduction in the expression of CAMP, leading to a weakened innate and adaptive immune response. Mechanistically, several signaling pathways may be involved in the inhibition of VDR expression following ZIKV infection, and further work is necessary to determine the mechanism by which ZIKV inhibits VitD3 signaling through decreased VDR transcription. Other studies have indicated that certain viruses can inhibit VitD3 signal transduction. For instance, Yenamandra et al. [67] reported that VDR mRNA and protein production were lower in EBV-transformed cells than in primary B cells. Moreover, Gotlieb et al. [68] reported that HBV infection decreases the expression of VDR mRNA. Interestingly, it has been reported recently that HBV infection induces the expression of miR-125a, decreasing the levels of VDR mRNA and protein [69]. The authors concluded that downregulation of hepatic VDR expression by HBV/miR-125a is negatively associated with liver inflammation and fibrosis in patients with chronic HBV infection. In agreement with these results, we previously reported that ZIKV infection was able to induce the expression of miR-125a in macrophages [70], highlighting the role of miRNA-125a in the control of the VitD3 signaling pathway. We therefore hypothesize that ZIKV induces miRNA-125a expression, which, in turn, downregulates the expression of VDR. Furthermore, VDR gene variations have been suggested to correlate with chronic HBV infection [71]. In agreement with our results, Rieder et al. [64] showed a rapid, pronounced, and sustained downregulation of the VDR gene by CMV infection in mammalian cells.

VDR expression is modulated by TLRs, leading to the induction of CAMP mRNA expression [72]. The endosomal receptors TLR7 and TLR8 are responsible for recognizing single-stranded RNA that is rich in U or GU residues. These receptors play a crucial role in the identification of viral pathogens by activating the innate immune response, leading to the production of type I IFN [73]. Previous reports have shown that VitD3 reduces TLR7 mRNA expression in PBMCs from patients with systemic lupus erythematosus [74] and also reduces TLR8 mRNA expression in monocytes [75]. Although ZIKV infection downregulates TLR7 and TLR8 mRNA expression in human monocytes [45], we did not observe any effect of VitD3 treatment on TLR7 and TLR8 mRNA expression in either ZIKV-Mon or ZIKV-VitD3-Mon when compared to VitD3-Mon. Reduced levels of RIG-I and TLR8 mRNA expression have been observed in peripheral blood from patients in the acute phase of ZIKV infection [12]. The authors of that study hypothesized that reduced expression of RIG-I and TLR8 during ZIKV infection could be an escape mechanism used by the virus to evade the innate immune response. Martinez Viedma and Pickett [76] studied the behavior of ZIKV infection in human placenta (JEG-3) and human microglia (HMC3) cell lines and found that the TLR7/8 pathway was significantly inhibited in HMC3 cells, whereas it was activated in JEG-3 cells during viral infection [76]. That study showed that the antiviral response during ZIKV infection is highly dependent on the type of host cell being infected. One study showed that R848, a synthetic agonist of TLR7/8, inhibits ZIKV replication in monocytes and macrophages through induction of viperin protein synthesis [50]. Campbell and Spector [77] reported that activation of human macrophages with TLR8 agonists upregulates the expression of CYP27B1 and the VDR, leading to the induction of CAMP and inhibiting HIV-1 replication only in presence of VitD3-sufficient medium. Thus, a potential explanation for the inhibition of VDR and CAMP mRNA expression by ZIKV infection could involve the expression of innate immune receptors, such as TLR7 and TLR8, that recognize intracellular viral RNA. Both receptors are expressed in endosomes in monocytes, macrophages, myeloid dendritic cells, and regulatory T cells [78].

There is growing evidence that vitamin D plays important roles in modulating the innate immune response to viral infection and can suppress the inflammatory response [69, 79–81]. We hypothesized that VitD3 would attenuate the inflammatory response induced by ZIKV infection of monocytes. However, except for TNFα, whose peak observed at 12 hpi was significantly lowered by VitD3 treatment, our data showed that VitD3 treatment of human monocytes infected with ZIKV did not significantly affect the production of pro-inflammatory factors, including IL1β, IL6, IL10, CXCL8/IL8, CCL2, and CCL5. Thus, ZIKV may downregulate the immunomodulatory effects associated with VitD3 treatment. Significantly lower levels of TNFα have also been found in MDMs differentiated in the presence of VitD_3_ and infected with DENV-2, [23]. In a study by Khare et al. [82], it was also reported that pre-treatment with calcitriol significantly decreases IFN-β and TNFα expression levels in A549 cells infected with H1N1 influenza A virus. Increased levels of TNFα, IL6, and IL1β have been linked to the induction of fever and disease severity [83]. Anderson et al. [84] reported that treatment with VitD3 has a significant impact on cytokine responses when co-stimulating PBMCs with *Pneumococcus* or RSV. This is consistent with what has been observed in severe/hemorrhagic infections in patients infected with DENV, another flavivirus, eliciting a pro-inflammatory cytokine response involving IL6 and CXCL8/IL8 [85, 86]. Gui et al. [87] reported that VitD3 treatment reduces IL6 production in the earlier stages of H9N2 influenza virus infection in human lung A549 epithelial cells and in mice, but increases its expression in the later stage of infection.

It has been reported that chemokines play a significant role in protection against congenital Zika syndrome (CZS). For instance, higher levels of CXCL8/IL8 have been found in the cerebrospinal fluid of neonates without CZS who were born to mothers infected with ZIKV during pregnancy than in those born with CZS-related microcephaly [88]. CXCL8/IL8 is a crucial participant in several inflammatory processes [89], whereas CCL2 and CCL5 are regulated by the infiltration of inflammatory cells [90]. Interestingly, high levels of CCL2/MCP-1 have been reported in patients with acute DENV, ZIKV, CHIKV, DENV/ZIKV, or CHIKV/ZIKV infections [91]. In DENV/ZIKV- or CHIKV/ZIKV-coinfected patients, the levels of CCL2/MCP-1 and TNFα show a significant inverse correlation with the ZIKV viral load. CCL2/MCP-1 is a potent monocyte-attracting chemokine that is involved in the recruitment of blood monocytes to sites of inflammatory responses [92]. Furthermore, higher levels of CCL2/MCP-1 and TNFα expression have been observed in ZIKV-infected mothers who gave birth to infants with congenital malformations of the central nervous system than in pregnant women whose fetuses were normal [13]. In patients with acute ZIKV infection, high levels of CCL5/RANTES have been reported to be linked to specific clinical symptoms [93]. On the other hand, IL10 is involved in the reduction of inflammatory responses, antigen presentation, and phagocytosis [94], thus preventing tissue damage due to an exacerbated immune response [95]. Concentrations of pro-inflammatory and anti-inflammatory mediators, including IL1β and IL10, are elevated in comparison to controls in newborns with ZIKV-associated microcephaly [88], as has been shown previously by Tappe et al. [14].

In summary, although calcitriol has been shown previously to downregulate the inflammatory response and promote antiviral activity *in vitro*, in this study, VitD3 treatment following ZIKV infection of monocytes did not have a significant effect on viral replication or the inflammatory response to ZIKV infection. These observations provide novel insights that will be relevant for future studies investigating the anti-inflammatory and antiviral role of VitD3 during ZIKV infection and the effects of vitamin D metabolites on ZIKV infection/replication.

## Limitations of the study

While our interpretations are supported by the results, a significant limitation of the study is that dysregulation of the VitD3 pathway was assessed based entirely on changes in mRNA expression, which may not reflect the full impact of infection or treatment. Further studies are needed to assess differences in expression at the protein level in order to clarify how ZIKV regulates gene expression in infected monocytes. Nevertheless, our findings clearly suggest that ZIKV infection of monocytes results in changes in the transcription of various genes, in particular, those related to the VitD3 signaling pathway.

## Conclusion

In this study, vitamin D treatment of human primary monocytes did not suppress ZIKV replication or affect the inflammatory response. However, VDR, CYP24A1, and CAMP expression were downregulated in ZIKV-infected monocytes treated with vitamin D, suggesting that VDR is involved in the response to ZIKV replication.

### Supplementary Information

Below is the link to the electronic supplementary material.

Supplementary file1 (JPG 352 KB)
