# Supplementary figures and images for: Zika virus infection suppresses CYP24A1 and CAMP expression in human monocytes
Source: Arch Virol. 2024 Jun 6;169(7):135. doi: 10.1007/s00705-024-06050-2 (PMC11153301; doi:10.1007/s00705-024-06050-2)

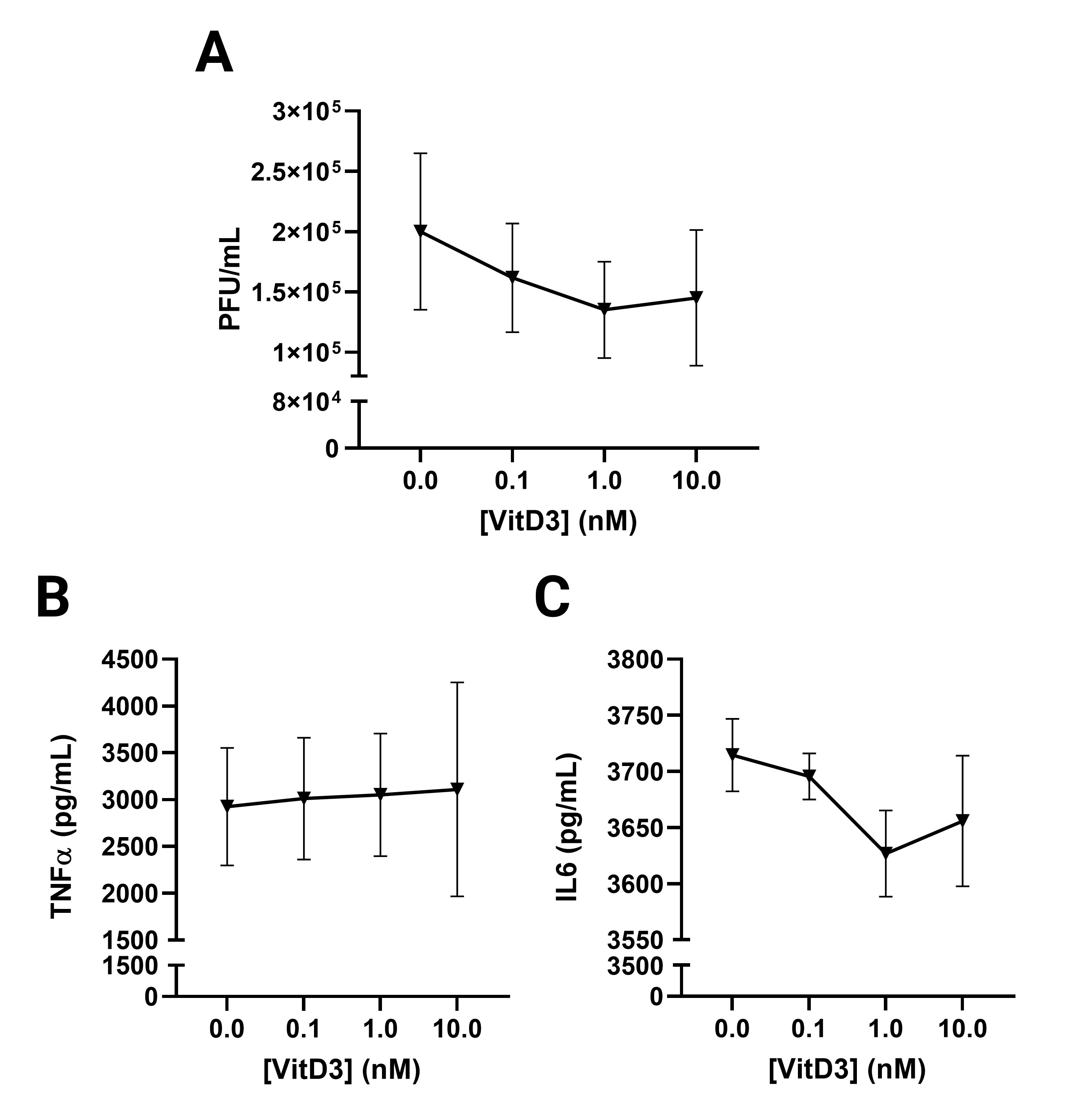

Supplement: Supplementary file 1 — Supplementary file1 (JPG 352 KB) [file 705_2024_6050_MOESM1_ESM.jpg]
